# Intraoperative Fluorescent Ureter Visualization in Complex Laparoscopic or Robotic-Assisted Gynecologic Surgery

**DOI:** 10.3390/jpm13091345

**Published:** 2023-08-31

**Authors:** Jiyoun Kim, Yoon Jang, Su Hyeon Choi, Yong Wook Jung, Mi-La Kim, Bo Seong Yun, Seok Ju Seong, Hye Sun Jun

**Affiliations:** 1 Department of Obstetrics and Gynecology, CHA Gangnam Medical Center, CHA University School of Medicine, Seoul 06135, Republic of Korea; acarcia74@chamc.co.kr (J.K.); b223023@chamc.co.kr (Y.J.); k345@chamc.co.kr (S.H.C.); sunghunpapa@naver.com (Y.W.J.); mila76@naver.com (M.-L.K.); sjseongcheil@naver.com (S.J.S.); 2 Department of Obstetrics and Gynecology, CHA Ilsan Medical Center, CHA University School of Medicine, Goyang 10414, Republic of Korea; bosunyun@chamc.co.kr

**Keywords:** indocyanine green, laparoscopic or robotic-assisted gynecologic surgery, near-infrared fluorescence imaging, real-time ureter

## Abstract

This study aimed to demonstrate the feasibility of ureteral navigation using intraoperative indocyanine green (ICG) and near-infrared fluorescence (NIRF) imaging during complex laparoscopic or robot-assisted gynecologic surgery (LRAGS). Twenty-six patients at high risk of ureteral injury with complex pelvic pathology (CPP) due to pelvic organ prolapse (POP), multiple myomas, large intraligamentary or cervical myoma, severe pelvic adhesions, or cervical atresia underwent LRAGS. All patients underwent cystoscopic intraureteral ICG instillation before LRAGS and ureteral navigation under NIRF imaging intraoperatively. Both ureteral pathways were identified from the pelvic brim downwards through NIRF imaging in all patients, even though some were not visualized under the white light mode. The fluorescent ureters were visualized immediately after the beginning of surgery and typically lasted for >5 h during surgery. There were no cases of iatrogenic ureteral injury. The hemoglobin decrement was 1.47 ± 1.13 g/dL, and no transfusion was required. In our study, both ureters in all patients were identified with ICG-NIRF imaging during LRAGS, and these techniques made surgeries easier and safer. Despite the CPP, there was no ureteral injury or transfusion following surgery. Further prospective studies are needed to introduce intraoperative ureteral guidelines for ICG-NIRF imaging during LRAGS with CPP.

## 1. Introduction

Iatrogenic ureteral injury (IUI) is reported to be the most common complication of major pelvic surgeries, occurring at rates of 0.35–1.5% [1]. During hysterectomy for severe endometriosis or high-risk reconstructive pelvic surgery for pelvic organ prolapse [2,3], this rate increases up to 7.6–11%, which leads to serious complications and legal problems if not detected during surgery. Therefore, preoperative double-J stents or a lighted ureteral stent are inserted to avoid ureteral damage [4]. However, this is expensive and difficult for gynecologists to perform. There are also procedural complications, such as perforation or thermal injury, and there is no evidence that stent insertion prevents ureteral damage in the gynecologic field. Therefore, their routine use remains controversial [5,6].

Minimally invasive surgery is gaining popularity in gynecologic surgery, but it lacks tactile feedback. Consequently, IUI is not uncommon during laparoscopic and robotic surgeries. If the retroperitoneal ureters of patients at high risk of ureteral injury could be visualized in real time, ureteral complications from complex pelvic surgery could be prevented.

Indocyanine green (ICG) is a cyanine-based water-soluble dye that fluoresces in the near-infrared (NIR) spectrum. It can be administered intravenously several times with a short lifetime and low adverse effects. The ICG-NIRF technique was first used clinically to measure hepatic function, cardiac output, and ophthalmic angiography after approval for intravenous administration by the FDA in 1959. In addition, since it can estimate the perfusion of tissues and organs in real time, its clinical application has increased in recent years to assess blood flow in colorectal anastomoses and urologic surgery [7]. Still later, fluorescence guided surgery has been progressively studied and applied to robotic or laparoscopic gynecologic surgery, such as sentinel lymph node biopsy in gynecologic malignancies, endometriosis mapping, and vascular perfusion of the ureters, vagina, or ovaries [8,9,10,11] Systemically administered ICG is secreted initially through the liver, bile duct, and intestines, not the kidneys, and so, it has to be administered in retrograde instillation by ureteral catheter for intraoperative ureter visualization [12]. Recently, the illuminative identification of ureters through intraureteral ICG instillation and near-infrared fluorescence (NIRF) imaging has been reported to be useful during robotic or laparoscopic surgery [7,13,14,15]. Cabanes et al. reported that this technique was applied to facilitate intraoperative ureteral dissection and accurately identify the ureteral pathway in real time in sixteen patients during gynecological oncology surgery; the surgeons could visualize both ureters and avoid ureteral injury in all patients [16].

This study was conducted to demonstrate the feasibility of ureteral navigation using intraureteric ICG and NIRF imaging during complex laparoscopic or robotic gynecologic surgery (LRAGS) to avoid iatrogenic ureteral injuries, simplify surgeries, and improve patient outcomes.

## 2. Materials and Methods

### 2.1. Patients

We performed ureteral monitoring through intraoperative ICG-induced NIRF imaging for 26 patients at high risk of ureteral damage due to complex pelvic pathologies or pelvic reconstructive surgery during LRAGS. We compared it with white light mode through video recording and analyzed the short-term outcomes. These techniques were performed by a single surgeon at CHA Gangnam Medical Center from September 2021 to August 2022. We performed robot-assisted total laparoscopic hysterectomy (TLH) and sacrocolpopexy (SCP), sacrohysteropexy, and TLH with uterosacral ligament suspension (USLS) due to pelvic organ prolapse. We performed robot-assisted TLH or myomectomy and TLH with pelvic anatomical distortion due to a large intraligamentary myoma, cervical myoma, multiple myomas, severe endometriosis, and pelvic adhesions, or severe cervical atresia after postmenopausal cone biopsy. Occasionally, single-port access TLH was carried out. These 26 patients underwent intraureteral ICG instillation into both ureteral orifices via a cystoscopic 6-F open-end catheter before LRAGS. A 6 mm hysteroscope (Stortz, Hopkins^®^ II) with an operating channel was used instead of a cystoscope for easy access by gynecologists. All procedures used the da Vinci Si or Xi robotic system (Intuitive Surgical, Inc., Sunnyvale, CA, USA) with integrated Firefly NIRF imaging or a laparoscopic camera system with conversion from white-light imaging to NIRF mode to detect the fluorescence of ICG (Stryker’s 1688 4 K Advanced Imaging Modalities [AIM] platform with SPY^®^ Fluorescence Imaging Technology).

The exclusion criteria were a history of allergic reactions to ICG or iodide and psychiatric medication use.

All patients received ICG (Diagnogreen, Cheil Pharmaceutical Co., Seoul, Republic of Korea) for this procedure. The study protocol was approved by the Institutional Review Board of CHA Gangnam Medical Center (GCI 2022-09-004); informed consent requirements for the study were waived given its retrospective nature.

### 2.2. Surgical Interventions

**Figure 1:**
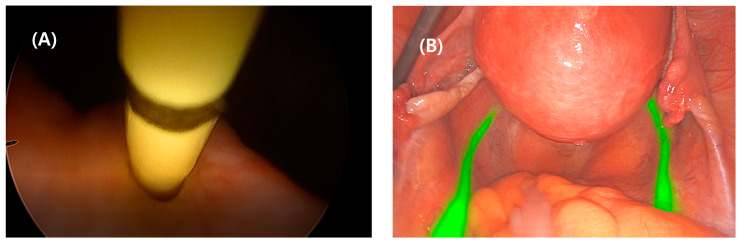
Cystoscopic view: intraureteral 6-Fr open-ended catheter insertion into the left ureteral orifice for indocyanine green (ICG) instillation (**A**). Laparoscopic bilateral ureteral visualization under ICG and near-infrared fluorescence (NIRF) imaging (**B**).

Prior to the robotic or laparoscopic surgery, a hysteroscope was inserted into the bladder, the tip of a 6-F open-end ureteral catheter was inserted into one of the ureteral orifices, 25 mg ICG was mixed with 10 cc of sterile water, 5 cc was instilled into the midureter, approximately up to 10 cm in the ureter, through the catheter, and the catheter was slowly withdrawn (Figure 1A). The remained 5 cc was inserted into the opposite ureter using the same procedure. At this time, the ICG stained the epithelial layer of the ureter and bladder rapidly and reversibly and did not extravasate unless excessive pressure during instillation. The catheter tip was placed on the ureteral orifice for 1 to 2 min to maximize the intraureteral ICG retention before complete catheter removal. During robotic or laparoscopic surgery, the da Vinci surgical robotic or laparoscopic NIRF laser-stimulated ICG molecules emitted near-infrared light, which turned green through the camera filter lens system. Thus, the green-fluorescent ureters were clearly visualized in real time in all 26 surgical patients. Even in some cases when the ureteral orifice was difficult to identify owing to severe pelvic pathology or ureteral atrophy, it could be easily detected by intravenous indigo carmine injection in advance. A guide-wire-assisted catheter insertion was more helpful for approaching the orifice. In addition, the entire ureter could be more clearly identified by lengthening the catheter withdrawal time.

All patients were assessed for (1) the time that the ureter was first viewed by ICG-NIRF imaging, (2) how far down from the pelvic brim both sides were visible, and (3) overall duration. Differences in clarity between the da Vinci and the laparoscopic system were analyzed.

## 3. Results

### 3.1. Baseline Characteristics

LRAGS was performed in 26 patients with complex pelvic pathology, and the ureter was successfully visualized in real time during the operation via ICG-NIRF imaging. The clinical characteristics of the patients are shown in Table 1. The mean age of the patients was 50.1 years (31–71 years). The most common preoperative diagnoses were multiple myomas (57.7%, *n* = 15) or pelvic organ prolapse (30.8%, *n* = 8), and the most frequent surgical procedure was TLH. The most important factors in patient selection were complex gynecologic pathology or pelvic reconstructive surgery, which were at high risk of ureteral injury during surgery.

The average BMI of the patients was 22.1 kg/m^2^ (range 17.7–25.7). Eleven patients (42.3%) had undergone previous surgery, including previous cesarean section (23.1%, *n* = 6), exploratory laparotomy (3.8%, *n* = 1), robotic myomectomy (3.8%, *n* = 1), laparoscopic surgery (7.7%, *n* = 2), and appendectomy (3.8%, *n* = 1). Among fifteen patients (57.7%) with pelvic adhesions, six (23.1%) had adhesions beyond moderate level.

### 3.2. Intraoperative Characteristics

The fluorescent ureter was clearly visualized in real time in all 26 patients (Figure 1B).

ICG-NIRF ureters were visible along the pelvic brim bilaterally. There were no distinctive differences in clarity between the da Vinci and laparoscopic systems. In seven out of twenty-six cases, ureteral peristalsis was nearly invisible in white light mode, it was possible by employing the ICG-NIRF imaging technology and unnecessary ureteral dissection was avoided. In cases where ureteral peristalsis detection was difficult in white light mode due to thick surrounding visceral fat, adhesion, and fibrosis or the presence of left rectosigmoid, persistent ureteral illumination via ICG-NIRF imaging was helpful. On average, the procedures for cystoscopic intraureteral ICG instillation took approximately 10–20 min.

**Figure 2:**
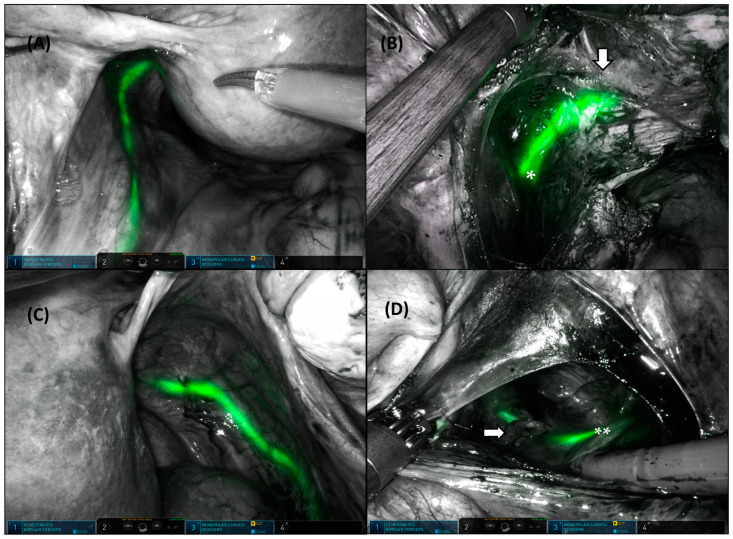
Bilateral fluorescent ureter identification during robotic-assisted total hysterectomy. Left ureter under ICG-NIRF imaging (**A**). Left uterine artery (bottom arrow) crossing over the left ureter (*) by lateral approach (**B**). Right ureter under the ICG-NIRF imaging (**C**). Right uterine artery (right arrow) crossing over the right ureter (**) by lateral approach (**D**).

Four cases had ureteral atrophy due to advanced age, which made the identification of the ureteral orifice difficult during cystoscopy. In these four cases, intravenous indigo carmine injection was carried out to visualize blue urine efflux prior to catheter insertion; alternatively, guided wire insertion was performed in advance to localize ureteral orifices, which allowed ICG injection without difficulty (Figure 1). In five cases of anticipated heavy bleeding during hysterectomy due to extremely large multiple myomas, bilateral uterine arterial ligation from a lateral approach to the parametrium was performed after ICG navigation of the ureteral pathway (Figure 2).

The fluorescence was visualized by illuminating the ureters immediately after the beginning of surgery following intraureteral ICG, and typically lasted for >5 h and disappeared within 8 h.

### 3.3. Surgical Outcomes

Table 2 summarizes the surgical outcomes. The mean operating time was 189.8 (120–375) minutes and the mean blood loss was 176.9 (100–500) mL. Hemoglobin decrement was 1.47 ± 1.13 mg/dL with mean preoperative hemoglobin of 12.7 mg/dL and mean postoperative hemoglobin of 11.3 mg/dL. There were no intraoperative or postoperative blood transfusions.

Postoperative complications included mild pulmonary edema for 1–2 days postoperatively in two patients with robotic TLH with SCP. Two patients with SCP were 70 years old and had underlying medical illnesses such as hypertension or arrhythmia, and the operation time was as long as 340 min. The mean postoperative hospital stay was 4.1 (range, 3–6) days. There were no intraoperative or postoperative complications of Clavien–Dindo grade III or higher. Side effects related to ICG were not observed for up to 2 months.

## 4. Discussion

The objective of this study was to demonstrate the feasibility of ureteral identification using an intraureteric ICG and NIRF imaging system during LRAGS for patients at high risk of ureteral injury due to complex pelvic pathologies to prevent iatrogenic ureteral injuries and facilitate surgeons’ decision-making processes.

More than half of ureteral injuries occur during pelvic surgery [17,18,19] and most are recognized postoperatively and subsequent to serious complications such as urologic fistula, loss of renal function, reoperation, and legal problems [1,2]. Prophylactic double-J stent or lighted stent insertion for the prevention of ureter injury are considered controversial and require urologists’ assistance [4]. Furthermore, complications of the procedure itself, such as ureteral perforation, avulsion, incorrect passage, and thermal injury, have been reported [5,6].

Recently, robotic or laparoscopic surgery has been preferred due to its cosmetic advantages and rapid recovery; however, it has no or insufficient haptic feedback [18,19]. Therefore, if there is an adjuvant technological imaging system for precise visualization of the retroperitoneal ureter, iatrogenic ureteral damage can be avoided, surgery can become safer and can provide reassurance to surgeons, and even beginners can reduce the learning curve through ureteral navigation [20].

Lee et al. first reported intralumenal ICG injection of the ureter during a ureteroureterostomy for intraoperative ureter visualization [6]. Siddighi et al. showed definitive ureter delineation in more than 10 robotic-assisted laparoscopic SCP patients [5]. Mandovra et al. reported 30 cases of laparoscopic gynecologic and non-gynecologic surgeries, and White et al. reported 16 cases of complex colorectal surgery [21,22]. The findings of the present study correspond well with those of previous reports.

Soriano et al. compared intraureteric ICG injection with or without ureteral stent placement for intraoperative ureter identification in 83 patients during robotic colorectal resections. They demonstrated that cystoscopy and ICG injection alone is faster and safer than when combined with catheter placement. There was no difference in visualization between injection alone and stent indwelling [23]. Mahalingam et al. suggested a novel near infrared fluorescent dye (UreterGlow) that can be administered intravenously but is excreted primarily through the renal system and can facilitate intraoperative ureter illumination for more than 2 h in pigs [12]. Slooter et al. described the currently available and experimental dyes for intraoperative ureter identification [24]. New drugs for this technology have been investigated to visualize ureters in fluorescence through ongoing clinical trials [7].

There have been no significant toxic effects from ICG in humans even with a high dose of 5 mg/kg of body weight, and severe complications related to ICG are rare. Chu et al. once reported a case of life-threatening anaphylactic response after intravenous ICG administration during robotic partial nephrectomy [25].

The strength of this study was that it performed accurate real-time visualization of ureter delineation through intraureteral ICG injection in twenty-six patients at high risk of ureteral injury during laparoscopic or robot-assisted gynecologic surgery. Despite our small sample size, this is the largest number of cases among benign complex gynecologic surgeries to date with this technique. Even if some cases were not detected in white-light mode, all were confirmed via ICG-NIRF imaging. Second, despite the complex uterine pathologies, no transfusions were required, because blood loss was minimized by avoiding unnecessary dissection around the ureter due to its visualization. This technique was most helpful for minimizing blood loss in bilateral uterine arterial ligation after ureter identification from a lateral approach to the parametrium [26]. Third, a hysteroscopic instrument familiar to gynecologists was used for intraureteral ICG injection instead of cystoscopy, which was easily prepared without urologists’ assistance upon encountering an intraoperative obstacle. Fourth, through intraluminal ICG, it was possible to visualize the ureter more precisely and in prolonged real time until the surgeries were completed. Fifth, because this study was performed by a single surgeon, interindividual variations by surgical experience were minimized.

Our study had some limitations. First, the additional time, cost, and an NIRF imaging camera system to detect the fluorescence of ICG were needed for this procedure. This can increase the complexity of the surgery. Second, the excessive use of imaging technology could interfere with surgical training. Third, this analysis was limited to a small number of cases performed by a single surgeon, thus limiting the generalizability of our conclusions. Therefore, it is necessary to increase the evidence level by applying it to larger populations in multiple centers. In addition, this technique should also be applied to patients with severe endometriosis, gynecologic malignancy, or congenital anomalies, which were reported by Ianieri et al. but lacking in this study [16,27,28]. Fourth, intraureteral instillation is an off-label use compared to intravenous injection of ICG, which has already been approved for use by the FDA; therefore, safety should be proven in more cases. Since the excellent safety of intravenous ICG has been established [25], there may be fewer anaphylactic reactions because intraluminal injection is more topical. This work was based on the analysis of different types of interventions for avoiding iatrogenic ureteral injury during surgery, which have different risks (some very low, others high) of causing damage to the ureteral course even though risk is rare. Despite these limitations, the additional cystoscopic ICG procedure is a relatively easier, cheaper, safer, and much less time-consuming option than other alternatives for intraoperative ureter identification compared with a potential ureteral injury. Therefore, the application of this promising technology for preventing ureteral injury during gynecologic surgery should be expanded to reduce morbidity of patients and to provide reassurance to the surgeons.

## 5. Conclusions

In our study, intraureteral ICG-NIRF imaging could identify both ureters in real time during surgery despite the presence of complex pelvic pathology. This imaging technology could prevent iatrogenic ureteral injury and minimize bleeding following surgery without ureteral dissection. Taken together, this technology is simple, inexpensive, and reproducible for intraoperative ureteral detection, and could also help surgeons’ decision-making and provide psychological reassurance during the operation. Nonetheless, adding an intraureteral ICG-NIRF imaging procedure could lengthen operation time. To be introduced as a universal guideline for complex gynecological operations at high risk of ureteral injury, it would be necessary to first discuss the effectiveness and risk–benefit analysis through several randomized controlled trials and systematic reviews.

## Figures and Tables

**Table 1:** Clinical characteristics.

Characteristics	Values
No. of patients	26
Age, years (range)	50.5 (31–71)
Body mass index, kg/m^2^ (range)	22.1 (17.7–25.7)
Patients with prior abdominal or pelvic surgery, *n* (%)	11 (42.3)
Cesarean section	6 (23.1)
Explo-laparotomy	1 (3.8)
Robotic myomectomy	1 (3.8)
Laparoscopic surgery	2 (7.7)
Appendectomy	1 (3.8)
Preoperative diagnosis, *n* (%)	
Pelvic organ prolapse	8 (30.8)
Large or multiple or intraligamentary or cervical myomas	15 (57.7)
Adenomyosis and endometriosis	2 (7.7)
Severe cervical stenosis and cervical intraepithelial neoplasia 2	1 (3.8)
Type of surgical procedures, *n* (%)	
Robotic sacrohysteropexy with AP *** repair	3 (11.5)
Robotic assisted TLH * and sacrocolpopexy with posterior repair	2 (7.7)
TLH with USLS ** with AP *** repair	3 (11.5)
Robotic assisted TLH *	2 (7.7)
TLH *	12 (46.2)
Robotic myomectomy	4 (15.4)

* TLH: Total laparoscopic hysterectomy. ** USLS: uterosacral ligament suspension. *** AP: anterior and posterior. Values are presented as number of patients (%). Values are presented as the mean (range).

**Table 2:** Surgical outcomes.

Surgical Outcomes	Values
Visibility of ureter by indocyanine green, *n* (%)	26/26 (100%)
Estimated blood loss, mL (range)	176.9 mL (100–500)
Change in hemoglobin, mg/dLL	1.4 ± 1.1 mg/dL ^(a)^
Total operative time, minutes (range)	189.8 mim (120–375)
Postoperative hospital stay, days (range)	4.1 (3–6)
Postoperative complication, *n* (%) ^(b)^	0 (0%)

Values are presented as number of patients (%). Values are presented as the mean (range) or mean ± standard deviation ^(a)^. ^(b)^ Clavien–Dindo grade III or higher.

## Data Availability

Data will be available upon reasonable request from the corresponding author. However, the data cannot be made public to maintain women’s privacy and for legal reasons, as it contains private health information along with identifiers.
